# Supplementary material for: iPSC-Derived Cardiomyocytes in Inherited Cardiac Arrhythmias: Pathomechanistic Discovery and Drug Development
Source: Biomedicines. 2023 Jan 25;11(2):334. doi: 10.3390/biomedicines11020334 (PMC9953535; doi:10.3390/biomedicines11020334)
Supplement: Supplementary file 1 [file biomedicines-11-00334-s001.zip › biomedicines-2152013-supplementary.pdf]

Supplementary Table S1: overview of previously published 2D iPSC-CM cardiac arrhythmia disease models

Syndrome	Causal gene	Experimental approach	Cellular phenotype	Ref
LQTS	*KCNQ1* (c.1893del)	PC, MEA	Reduced I_{Kr}, prolonged APD, reduced wild-type *KCNQ1* mRNA and protein	[102]
	KCNQ1 exon 7 deletion	PC	Reduced I_{Ks}, APD prolongation, reduced wild type *KCNQ1* mRNA and protein, small molecule ML277 partially restored APD and reversed the decreased I_{Ks}.	[103]
	KCNQ1 p.(Arg594Gln) p.(Arg190Gln)	PC, MEA	Prolonged APD, reduced I_{Ks} activation that was reversed by hERG allosteric modulator LUF7346	[104]
	KCNH2 p.(Ala614Val)	PC	Prolonged APD, reduction of I_{Kr}, EADs, arrhythmias and potential improvement with pinacidil	[105]
	KCNH2 p.(Arg176Trp)	PC, MEA	Prolonged APD, reduced I_{Kr}, demonstrated arrhythmogenic electrical activity	[106]
	KCNH2 p.(Gly1681Ala)	PC	APD prolongation and EADs	[92]
	KCNH2 p.(Asn996Ile)	PC, MEA	Prolonged APD, reduced I_{Kr} activation that was reversed by hERG allosteric modulator LUF7346	[104]
	SCN5A p.(Phe1473Cys)	PC	Delayed repolarization, prolonged QT interval with increase in pacing improving the phenotype, increased risk of fatal arrhythmia	[107]
	SCN5A p.(Val1763Met)	PC	Prolonged APD, elevated late I_{Na} current, $Na_v1.5$ blocker can reverse related symptom	[108]
	SCN5A p.(Val240Met) p.(Arg535Gln)	PC	Insignificant increase in APD, delayed time to peak I_{Na} inactivation	[109]
	SCN5A p.(Arg1644His)	PC, MEA	Prolonged APD, high EADs, and accelerated recovery from inactivation of Na^+ currents. Rescue of abnormal phenotypeby mexiletine and ranolazine	[103]

Syndrome	Causal gene	Experimental approach	Cellular phenotype	Ref
	KCNJ2 p.(Arg218Trp) p.(Arg67Trp) p.(Arg218Gln)	MEA, CI	Strong arrhythmic events, higher incidence of irregular Ca^{2+} release. Flecainide, but not pilsicainide, suppressed irregular Ca^{2+} release and arrhythmic events	[111]
	CACNA1C p.(Gly1216Ala)	PC, CI	APD prolongation and DADs, abnormal calcium handling, irregular and slow contraction. Roscovitine rescued abnormal cellular phenotype	[112]
	CALM1 p.(Phe142Leu)	PC, MEA, CI	Prolonged APD, defective $I_{Ca\text{-}L}$ inactivation, altered rate-dependency and response to isoproterenol. Repolarization abnormalities reversed by verapamil	[113]
	CALM2 p.(Asn98Ser)	PC	Lower beating rate, prolonged APD, and impaired $I_{Ca\text{-}L}$ inactivation, correction of the mutant allele rescued abnormal phenotype	[114]
	CALM2 p.(Asp130Gly)	PC, IF	Prolonged APD, disrupted Ca^{2+} cycling properties, and diminished Ca^{2+}/CaM-dependent inactivation of $I_{Ca\text{-}L}$. Suppressing the mutant gene rescued abnormal phenotype	[115]
	PKP2 (c.2484C>T)	PA, MEA, CI	Reduced I_{Na}, deficit restored by transfection of WT gene	[116]
	PKP2 p.(Arg101His)	PC	Reduced APD90	[84]
BrS	*RRAD* p.(Arg211His)	PC, CI	Reduced Vmax of AP, prolonged APs and increased incidence of EADs, decreased I_{Na} peak amplitude, increased I_{Na} persistent amplitude, decreased $I_{Ca\text{-}L}$ amplitude	[117]
	SCN5A p.(Arg1638X) p.(Trp156X)	PC	Reduced Vmax, reduced I_{Na}	[118]
	SCN5A p.(Ala226Val)+ p.(Arg1629X) *SCN5A*	PC	Ala226Val/Arg1629X: Reduced I_{Na}, reduced Vmax and APA Thr1620Met: no effect on I_{Na} and normal AP	[119]

Syndrome	Causal gene	Experimental approach	Cellular phenotype	Ref
	p.(Thr1620Met)			
	SCN5A p.(Arg367His)	PC	Reduced I_{Na}, shift in activation and inactivation voltage-dependence curves, faster recovery from inactivation	[120]
	SCN5A p.(Ala735Val)	PC	Reduced APA and Vmax, reduced I_{Na}, shift in activation and inactivation voltage-dependence curves	[121]
	SCN10A p.(Arg1250Gln)+ p.(Arg1268Gln)	PC, CI	Reduced peak I_{Na} and I_{NaL}, accelerated recovery from inactivation in patient iPSC-CMs, reduced I_{Ca-L} and I_{Ks}, reduced APA and Vmax, increased EAD-like events	[85]
	SCN1B p.(Leu210Pro)+ p.(Pro213Thr)	PC, CI	Reduced peak I_{Na} and I_{NaL}, positive shift in the voltage dependence of activation and negative shift of the inactivation, reduction in IKs and I_{Kr}, Reduced APA and Vmax, increased arrhythmia like events	[86]
CPVT	*RYR2* p.(Ser406Leu)	PC, CI	Elevated diastolic Ca^{2+} concentrations, a reduced SR Ca^{2+} content, DADs and arrhythmia, dantrolene can restore these phenotype	[122]
	RYR2 p.(Pro2328Ser)	PC, CI	Increased non-alternating variability of Ca^{2+} transients in response to isoproterenol and β-agonists decreased AP upslope velocity	[122]
	RYR2 p.(Met4109Arg)	PC, MEA, CI	DADs were eliminated by flecainide and thapsigargin	[123]
	RYR2 p.(Leu3741Pro)	CI, MEA	Altered intracellular Ca^{2+} homeostasis, β-adrenergic stimulation potentiated spontaneous Ca^{2+} waves and prolonged Ca^{2+} sparks. Flecainide ameliorated disease phenotype	[124]
	RYR2 p.(Ile4587Val)	PC, CI	Increased diastolic Ca^{2+} waves and DADs with pacing, while S107 suppressed the DADs	[126]

Syndrome	Causal gene	Experimental approach	Cellular phenotype	Ref
	CASQ2 p.(Asp307His)	PC, CI	β-adrenergic agonist caused DADs, oscillatory arrhythmic pre-potentials, and diastolic $[Ca^{2+}]_i$ rise	[35,126]
	CASQ2 p.(Asp307His)	PC, CI	Ca^{2+} transient irregularities, EADs, and reduced threshold for store overload-induced Ca^{2+} release, β-blockers prevented arrhythmia	[127]
ACM	*PKP2* (c.2484C>T) *PKP2* (c.2013delC)	CI, seahorse metabolic assay	Abnormal plakoglobin nuclear translocation, decreased β-catenin activity, exaggerated lipogenesis and apoptosis calcium-handling deficits	[128]

Adapted and updated from Garg et al. (2018) and Pan et al. (2021) [9,10]. PC: patch clamp; IF: immunofluorescence; MEA: Multi electrode array; WB: Western Blot; CI: Calcium imaging; AFM: atomic force microscopy; AP: action potential; I_{Ks}: slow delayed rectifier K+ current; ER: endoplasmic reticulum; APD50-90: Action potential duration at 50%–90% of repolarisation; EAD: early after depolarisation; I_{Kr}: rapid delayed rectifier K+ current; I_{Ca-L}: L-type calcium current; APA: action potential amplitude; Vmax: maximum rate of rise of the action potential; I_{Na}: sodium current; DAD: delayed after repolarisation; FP: field potential; CV: conduction velocity; I_{to}: transient outward current; SR: sarcoplasmic reticulum; EM: electron microscope.
